# Improving Recognition of Heart Failure With Preserved Ejection Fraction in the Outpatient Setting: A Pilot Study of Readily Useful Clinical Attributes

**DOI:** 10.7759/cureus.94159

**Published:** 2025-10-08

**Authors:** Khalid Sawalha, Aakash Rana, Lana Alamat, Andrew J Fancher, Munes Albadaineh, Angel Lopez-Candales, Talal Asif

**Affiliations:** 1 Cardiovascular Disease, University of Arkansas for Medical Sciences, Little Rock, USA; 2 Cardiometabolic Medicine, University of Missouri Kansas City School of Medicine, Kansas City, USA; 3 Internal Medicine, Central Arkansas Veterans Healthcare System, Little Rock, USA; 4 Internal Medicine, Jordan Hospital, Amman, JOR; 5 Internal Medicine, University of Kansas School of Medicine - Wichita, Wichita, USA; 6 Cardiovascular Medicine, University Health Truman Medical Center, Kansas City, USA; 7 Cardiovascular Disease, Saint Luke's Mid America Heart Institute, Kansas City, USA

**Keywords:** chart review, clinical phenotypes, echocardiography, heart failure with preserved ejection fraction, hfpef, outpatient clinic

## Abstract

Background: Heart failure with preserved ejection fraction (HFpEF) is increasingly acknowledged as a major clinical condition, yet its diverse presentation often hinders prompt diagnosis and management. This study aims to identify clinical variables obtainable during routine outpatient visits that can help in recognizing subclinical and unrecognized HFpEF or those at risk of developing it.

Methods: This retrospective study analyzed patient data from July 2022 to June 2023 at The University Health Truman Medical Center in Kansas City, Missouri, United States. Chart reviews were performed for patients seen in various outpatient clinics, focusing on those presenting with specific symptoms and comorbidities indicative of HFpEF. Data collected included demographics, vital signs, comorbidities, laboratory values, and echocardiographic findings.

Results: A total of 174 patients (mean age 60 ± 11 years) were included. The majority were female (53%) and White patients. Common comorbidities included hypertension, obesity, diabetes, and chronic kidney disease. Echocardiograms revealed that 93.5% of patients had at least one abnormality, with increased left ventricle (LV) wall thickness in 59.8% and abnormal LV relaxation in 28.7%.

Conclusion: Symptoms such as dyspnea, exercise intolerance, exertional chest pain, and easy fatigability should prompt early referral to cardiology, particularly in middle-aged and older patients with the relevant comorbidities seen in this study. Incorporating these clinical indicators into electronic medical records can enhance early recognition and diagnosis of HFpEF, improving patient outcomes.

## Introduction

Heart failure (HF) is a common clinical entity [1]. Even though estimates of HF incidence in the United States continue to decline, the overall incidence of HF with preserved ejection fraction (HFpEF) specifically continues to increase [2-4]. The prevalence of this subtype of HF has been increasing steadily, and HFpEF now represents more than 50% of all current HF cases [4]. While this subtype is sometimes considered to be less severe, in reality, HFpEF outcomes are comparable to clinical outcomes seen in patients with HF with reduced ejection fraction (HFrEF) [5]. To aggravate matters even further, the heterogeneous phenotypic presentation among HFpEF patients hinders prompt recognition and delays accurate diagnoses [6,7].

Mischaracterization of the pathophysiology of HFpEF as simply diastolic dysfunction and increased left ventricle (LV) filling pressures has been a significant obstacle, hindering the proper conceptualization of this condition [8]. Due to its heterogeneous nature of presentation and non-specific symptoms, a high index of suspicion is required to trigger appropriate testing in a timely fashion [8,9]. Without this, HFpEF would largely remain underdiagnosed and underrecognized [10,11].

To improve recognition, healthcare providers should be aware of which coexisting comorbidities are associated with HFpEF so that their presence consequently prompts more careful evaluations intended to exclude potential mimickers resembling HFpEF while providing patients with an accurate diagnosis [12-16].

When most clinicians think of HFpEF, they think of abnormal LV relaxation with increased LV stiffness at rest, which could worsen significantly with increases in heart rate, limiting cardiac performance [17-21]. Even when this is present, we must remain cognizant that many multifactorial pathophysiological mechanisms contribute to and create different HFpEF phenotypic expressions [22,23]. Limited understanding of the varieties of HFpEF phenotypic expressions leads to a lack of proper recognition, which unfortunately contributes to the unrelenting progression of HFpEF [24].

We believe that providing guidance to the healthcare providers who refer patients to cardiologists on recognizing which elements and patient profiles in day-to-day practice should prompt further evaluation and concern for HFpEF may help address the underrecognition and mischaracterization of HFpEF. Accordingly, we conducted a study to collect clinical variables that can be easily obtained during routine outpatient clinical visits to identify which signs and/or symptoms are important for properly identifying patients who may have subclinical and unrecognized HFpEF or are at risk of developing it.

## Materials and methods

This is a retrospective chart review study of patients who presented to the endocrinology, sleep medicine, obesity, nephrology, and cardiology clinics at The University Health Truman Medical Center in Kansas City, Missouri, United States, from July 2022 to June 2023. The data were collected by a cardiometabolic fellow (KS), who was assigned to these different outpatient clinic rotations during his fellowship.

Adult patients aged ≥ 18 years who presented to the above-mentioned clinics with at least one symptom from Table 1 were included in the study. 

**Table 1:** Inclusion criteria comprised the presentation of at least one of these symptoms

Presenting Symptoms
Fatigue
Dyspnea on exertion
Exertional chest pain
Reduced exercise capacity

Data were also collected on demographic information, comorbidities, vital signs, lab values, and echocardiographic parameters as displayed in Table 2.

**Table 2:** Collected data

Vital Information
Age
Gender
Ethnic background
Vital signs and body mass index
Clinical Comorbidities
Hypertension
Type 2 diabetes mellitus
Obesity
Obstructive sleep apnea
Coronary artery disease
Atrial fibrillation
Chronic kidney disease
Chronic obstructive pulmonary disease
Anemia
Documented chronotropic incompetence
Pulmonary hypertension
Vitamin D deficiency
Documented malignancy
Prior cerebrovascular events
Laboratory Data
Vitamin D levels
Serum creatinine
Hemoglobin (Hgb)
Brain natriuretic peptide (BNP)
Thyroid-stimulating hormone (TSH)
High-sensitivity cardiac troponins (hs-cTpI)
Echocardiographic Data
Left atrial size
Pulmonary pressures
Left ventricular wall thickness
Left ventricular diastolic function
Left ventricular (LV) ejection fraction

Continuous variables were summarized as means ± standard deviations (SDs), while categorical variables were expressed as frequencies and percentages. Prevalence estimates were calculated for comorbidities and symptom burden. Exploratory analyses were conducted to assess potential associations between clinical variables and HFpEF-related phenotypes. By leveraging outpatient encounters across multiple subspecialty clinics, this study was uniquely positioned to capture a diverse group of patients with overlapping cardiometabolic and systemic risk factors. This approach increases the likelihood of identifying individuals with unrecognized or early HFpEF phenotypes, thereby broadening the understanding of clinical variables that may signal disease risk outside traditional cardiology settings.

## Results

A total of 174 patients with a mean age of 60 ± 11 years and a mean BMI of 37.2 ± 11.4 kg/m^2^ were seen in the outpatient clinic for a routine follow-up appointment in the study period. As seen in Table 3, most were female (53%) and White (48.9%) patients.

**Table 3:** Demographical characteristics of the study participants (N=174)

Parameters	Frequency (Percentage)
Sex	
Female	92 (53%)
Male	82 (47%)
Race	
White	85 (48.9%)
Black	73 (42.0%)
Hispanic	10 (5.7%)
Asian or Pacific Islander	6 (3.4%)

Of the vital signs obtained, mean systolic (140 ± 20.7 mmHg) and diastolic (81 ± 9.8 mmHg) blood pressure readings, with a range of 98-212 mmHg/53-112 mmHg, were seen. The mean heart rate was 80 ± 12.8 beats per minute with a range of 55-110 beats per minute. Of the comorbidities studied, hypertension (93.1%), obesity (87.4%), diabetes (83.3%), and chronic kidney disease (78.2%) were the most prevalent conditions as displayed in Table 4. 

**Table 4:** Prevalence of comorbidities in the study participants (N=174) COPD: chronic obstructive pulmonary disease

Comorbidity	Frequency (Percentage)
Hypertension	162 (93.1%)
Obesity	152 (87.4%)
Diabetes	145 (83.3%)
Kidney Disease	136 (78.2%)
Sleep Apnea	92 (52.9%)
Anemia	78 (44.8%)
Coronary artery disease	52 (29.9%)
COPD	49 (28.2%)
Atrial fibrillation	25 (14.4%)
Pulmonary Hypertension	17 (10.3%)

Even though cardiac markers have been useful in identifying HFpEF patients, natriuretic peptide levels were measured in only 46.6% (n=121) of all participants, with levels elevated in 63% (n=76) of those cases. Similarly, high-sensitivity cardiac troponin was measured in 44.3% (n=115) of cases, with levels averaging 22.6 ng/L ± 17 (range, 5.6-39.6) in those who had it measured.

Finally, 154 (89%) patients had an echocardiogram. Of these patients, at least one echocardiographic abnormality was present in 144 (93.5%) patients, as shown in Table 5. LV ejection fraction ranged from 45% to 70%, with the largest group of 62 (35.6%) patients falling within the 55-60% range. LV wall thickness was increased in 104 (59.8%) patients; LV diastolic function assessment showed an abnormal relaxation in 50 (28.7%) patients, and 14 (8.0%) patients with pseudo-normal or restrictive patterns. Furthermore, left atrial size was normal in 129 (74.1%) patients, and pulmonary pressures were elevated in only 24 (13.8%) patients (Table 5).

**Table 5:** Echocardiographic variables of the study participants (N=174) LVEF: left ventricular ejection fraction; LV: left ventricle/ventricular

Echocardiogram Variables	Frequency	Percentage
LVEF (%)		
45-50	6	3.4%
50-55	16	9.2%
55-60	62	35.6%
60-65	50	28.7%
65-70	20	11.5%
No ECHO Available	20	11.5%
LV Wall Thickness		
Normal	50	28.7%
Increased	104	59.8%
No ECHO Available	20	11.5%
LV Diastolic Function		
Could not be assessed	45	25.9%
Normal	44	25.3%
Early relaxation	50	28.7%
Pseudo-normal (grade 2) or Restrictive (grade 3)	14	8.0%
No ECHO Available	20	11.5%
Left Atrial Size		
Normal	129	74.1%
Dilated	24	13.8%
No ECHO Available	20	11.5%
Pulmonary Pressure		
Normal	129	74.1%
Elevated	24	13.8%
No ECHO Available	20	11.5%

## Discussion

The primary aim of this study was to collect clinical variables, obtained during routine outpatient clinical visits, to elucidate which could be most useful in identifying patients with subclinical and unrecognized HFpEF or who are at risk of developing HFpEF. We identified that female and White patients, and those with hypertension, obesity, diabetes, and chronic kidney disease, appear to be at greater risk of having or developing HFpEF, as defined by published guidelines [25]. Similar findings have been reported by other investigators [26].

In our study, the use of cardiac biomarkers was limited to patients previously admitted to the hospital with HF symptoms. However, these cardiac biomarkers were not otherwise routinely used in the outpatient setting for routine follow-up.

Similarly, echocardiography was most frequently obtained during a prior admission and not routinely used in follow-up. We also realized retrospectively that reports at our facility were not comprehensive enough to provide all necessary recommended information for the most accurate assessment of LV diastolic function, critical to the evaluation of HFpEF [27]. These two limitations lead us to the realization that HFpEF may be underrecognized in both cardiology and primary care clinics and that improvements are needed to expand echocardiographic assessments to provide more accurate interpretations of patients with HFpEF whose underlying pathophysiology lies beyond reduced ejection fraction and valvular abnormalities.

Significant clinical challenges exist in recognizing HFpEF, resulting in the development of two diagnostic scoring tools to aid healthcare providers in the noninvasive diagnosis of HFpEF: the Heart Failure Association Pre-test assessment, Echocardiography and natriuretic peptide, Functional testing, Final etiology (HFA-PEFF) and the H2FPEF (Heavy, 2 or more Hypertensive drugs, atrial Fibrillation, Pulmonary hypertension, Elderly age > 60, elevated Filling pressures) scoring systems [28-31]. Although these tools are a step in the right direction, it is important to recognize some potential limitations. First, significant intrinsic discrepancies exist between scoring systems; hence, scores might not be interchangeable [32]. Second, these scoring tools have been primarily used at specialized HF centers; therefore, their practicality and most importantly, their overall utility in day-to-day nonspecialized clinical practices have not been validated [33] Third, most recently, data from Ariyaratnam et al. demonstrated in a cohort of 120 patients in whom the gold standard invasive diagnosis of HFpEF was used as the ultimate discriminator, HFA-PEFF and H2FPEF scores only demonstrated moderate accuracy in patients with atrial fibrillation [34].

For these reasons, we did not include into our study a head-to-head comparison between these HFpEF scoring tools and our proposed clinical profile analysis. Although these algorithms could be extremely useful in the clinic setting, our main intention was to identify other clinical indicators that could be more user-friendly for non-cardiologists in recognizing potential HFpEF patients. Most importantly, our main goal was to use these variables and incorporate them into electronic medical records, as potential clinical reminders, similar to current efforts used for identifying undiagnosed patients at risk of having transthyretin amyloidosis (ATTR) cardiomyopathy [35]. Furthermore, it can be argued that other recommendations have been provided by the American College of Cardiology. Specifically, in a recent 2023 guideline publication, a simplistic 13-point summary for managing HFpEF was released [36]. However, we also recognize that most of these publications and their findings are likely to remain outside the radar of many cardiologists and certainly most non-cardiologists. Consequently, we decided to use a simplistic approach that would start by asking specific questions to facilitate recognition of specific signs and/or symptoms to be used in conjunction with a set of demographics, vital data, comorbidities, and echocardiographic criteria to simplify recognition of potential patients with HFpEF. It is worth mentioning that our approach was implemented not solely in cardiology clinics but in other specialized non-cardiology clinics where clinical emphasis was different. We found that this approach was helpful in enhancing patient identification and referral to cardiology while increasing the number of additional tests. In addition, as previously mentioned, this study can provide cardiologists with useful feedback to provide more comprehensive diastolic LV function assessments. Finally, our efforts can also prove useful in the training of cardiometabolic fellows to be more aware and discriminative in the recognition and evaluation of potential HFpEF patients.

Despite the useful clinical information this simple analysis provided, we recognize the following study limitations. First, the retrospective nature of the analysis. This type of analysis has inherent biases and limitations associated with data collection and documentation. Second, the study was conducted at a single, safety-net institution, limiting the generalizability of our findings to other populations or settings. Third, the small sample size may not fully capture the entire spectrum of HFpEF presentations and characteristics, potentially affecting our conclusions. However, the purpose of this study was to provide a feasible system to implement into the electronic medical record that would alert healthcare providers of potential patient characteristics that would trigger more analysis, additional testing, and referral to cardiology to shorten the time to potential diagnosis. Fourth, a lack of invasive gold standard procedures that could have been ultimately used for confirmation of HFpEF diagnosis. Unfortunately, not all institutions have the capability to perform rest and provoked hemodynamics, employ provocation maneuvers, use vasoactive agents, or perform metabolic evaluations on a routine basis [37]. However, it is also important to understand that none of these targeted invasive diagnostic tests and procedures has been so far successful in decreasing the mortality burden of HFpEF [37]. Despite these limitations, our aim was to identify these patients through noninvasive hemodynamic testing so that treatments and comorbidities treatment can be initiated promptly and effectively [38-42]. Finally, we acknowledge that some patients were lost to follow-up before a definitive diagnosis could be established. Nevertheless, those who remained under observation received a presumptive diagnosis of HFpEF to ensure timely and appropriate treatment.

We believe that incorporating these patient risk profiles into our electronic medical record to prospectively compare them to the proposed H2FPEF or HFA-PEFF scoring systems is the most appropriate direction for future studies. This should facilitate recognition of clinical risk features by every healthcare provider to ultimately improve early referral and earlier diagnosis. The main intent of this study was to enhance patient recognition before an initial HF hospitalization occurred and provide a tool to enhance recognition of specific clinical phenotypes that would raise clinical suspicions regarding the potential of HFpEF among non-cardiologists, so that appropriate referrals can occur. At our institution, this opened the doors to refer HFpEF patients to our HF clinics, traditionally limited to primarily seeing HFrEF patients. Finally, our analysis allowed for better patient discrimination that ultimately would enhance phenotypic HFpEF characterization [43-45].

## Conclusions

Our retrospective analysis indicates that looking for symptoms of dyspnea, exercise intolerance, and easy fatigability is a great starting point for diagnosing HFpEF, particularly among middle-aged and older patients. Presence of these symptoms in females, obese individuals, hypertensive patients, diabetics, and patients with chronic kidney disease makes them particularly at risk and should lower clinicians’ threshold for early referral to cardiology or additional testing to exclude HFpEF.

Our study also suggests that providers should not disregard abnormal natriuretic peptide or high-sensitivity cardiac troponin levels, particularly when HFrEF and coronary artery disease have been excluded. Finally, although eventual confirmation of HFpEF might still require several tests, echocardiography remains a very useful initial test. Regarding this testing modality, it is important to recognize that aside from preserved LV systolic function, additional findings suggestive of HFpEF include very vigorous LVEF, increased LV wall thickness, dilated left atria, elevated pulmonary pressures, and grade 2 LV diastolic dysfunction. These findings are not only highly suggestive of HFpEF but also indicative that additional phenotypic characterization might be required.
